# Building a research registry for studying birth complications and outcomes in six Palestinian governmental hospitals

**DOI:** 10.1186/s12884-017-1296-6

**Published:** 2017-04-11

**Authors:** Sahar Hassan, Ase Vikanes, Katariina Laine, Khaled Zimmo, Mohammad Zimmo, Espen Bjertness, Erik Fosse

**Affiliations:** 1 Faculty of Nursing, Pharmacy and Health Professions and Institute of Community and Public Health, Birzeit University, Ramallah, State of Palestine; 2 The Intervention Centre, Oslo University Hospital, Oslo, Norway; 3 Department of Obstetrics, Oslo University Hospital, Ullevål, Oslo Norway; 4 Department of Obstetrics and Gynecology, Al Aqsa Hospital, Gaza, State of Palestine; 5 Department of Obstetrics and Gynecology, Shifa Hospitals, Gaza, State of Palestine; 6 Department of Community Medicine, Institute of Health and Society, University of Oslo, Oslo, Norway

**Keywords:** Childbirth, e-health, Birth registries, Case registration form, District Health Information Software, Completeness, Reliability, Government hospitals, Palestine

## Abstract

**Background:**

Electronic-health (e-health) provides opportunities for quality improvement of healthcare, but implementation in low and middle income countries is still limited. Our aim was to describe the implementation of a registration (case record form; CRF) for obstetric interventions and childbirth events using e-health in a prospective birth cohort study in Palestine. We also report the completeness and the reliability of the data.

**Methods:**

Data on maternal and fetal health was collected prospectively for all women admitted to give birth during the period from 1st March 2015 to 31st December 2015 in three governmental hospitals in Gaza and three in the West Bank. Essential indicators were noted in a case registration form (CRF) and subsequently entered into the District Health Information Software 2 (DHIS 2) system. Completeness of registered cases was checked against the monthly hospital birth registries. Reliability (correct information) of DHIS2 registration and entry were checked for 22 selected variables, collected during the first 10 months. In the West Bank, a comparison between our data registration and entry and data obtained from the Ministry of Health patient electronic records was conducted in the three hospitals.

**Results:**

According to the hospital birth registries, a total of 34,482 births occurred in the six hospitals during the study period. Data on the mothers and children registered on CRF was almost complete in two hospitals (100% and 99.9%); in the other hospitals the completeness ranged from 72.1% to 98.7%. Eighty birth events were audited for 22 variables in the three hospitals in the West Bank. Out of 1760 registrations in each hospital, the rates of correct data registration ranged from 81% to 93.2% and data entry ranged from 84.5% to 93.1%.

**Conclusions:**

The registered and entered data on birth events in six hospitals was almost complete in five out of six hospitals. The collected data is considered reliable for research purposes.

**Electronic supplementary material:**

The online version of this article (doi:10.1186/s12884-017-1296-6) contains supplementary material, which is available to authorized users.

## Background

After establishment of the Palestinian Authority (PA) in 1994, Palestinian health care has improved. Such improvements have included expansion of physical infrastructures [1], such as new hospitals and primary healthcare centers, increasing numbers of human resources and capacity building [2]. Additionally the Ministry of Health (MoH) recently applied an electronic health-information system that connects all governmental hospitals in the West Bank using AviCenna Health Information Medical System (www.avicenna-medical.com  [https://www.avicenna-medical.com/chronic-care-management-software-benefits-avitracks-cm], [3]). Electronic medical records (EMRs) are known to reduce medical errors by facilitating communication and retrieval of patient information. Easy access to patient health status reduces cost of healthcare due to less duplication of diagnostic tests and prescriptions of new medications. EMRs also provides data for research [4–6].

The MoH publishes annual reports on health status in Palestine mainly presenting information on coverage of health services, physical and human resources. However, these reports do not, comprise comprehensive health indicators reflecting the quality of health services or standards of care provided; for example, current indicators on maternal and newborn’s health include live births, delivery methods, birth weight but not gestational age at birth [7]. As part of the global Multiple Indicators Cluster Survey (MICS), the Palestinian Central Bureau of Statistics (PCBS) recently published national general indicators (*n =*13,964 women) on maternal health such as neonatal mortality rate, low birth weight, fertility rate, antenatal care coverage, birth coverage by skilled birth attendants, and institutional deliveries [8]. Despite that, we still lack national data on several internationally acknowledged indicators that are essential to understand the context and reflecting quality of care provided such as maternal body mass index, underlying medical disorders and therapeutic interventions associated with higher risk of adverse pregnancy outcomes.

Previous smaller hospital-based observational studies from Palestine have reported a liberal use of unnecessary interventions, such as routine use of episiotomy and limited compliance with use of international recommendations such as allowing mobility during labour and companionship [9, 10]. This has left Palestinian women to suffer from morbidities during and after childbirth that could have been avoided [11, 12]. Current knowledge on maternal and newborn health in Palestine may give a general understanding of its status, but is not sufficient to understand the specific processes, practices, or standards of care. This makes the evaluation of the quality challenging task and thus, improvements are slow or even not possible to be recommended or integrated.

Electronic health (e-health) is defined as “The use of information and communication technologies for health” [13–15]. E-health’s implementation in Low and Middle Income Countries (LMIC) is still limited. Although proven beneficial, it has been reported that the quality of data collected in LMIC has been inaccurate, unreliable and not timely [16]. Similar challenges have also been described from countries where medical and birth registries have been successfully implemented [4–6, 17]. Therefore, in order to identify gaps in processes of care there is a need to collect population based data in a prospective manner, also revealing details of routine care. Indeed, establishment of high quality birth registries has i.e. in the Scandinavian countries provided essential information to the Norwegian, Swedish and Danish authorities enabling them to apply evidence based changes for improvements of maternal-child health care [4, 18–20].

The objectives of the present study were to describe the implementation of a registration (case record form; CRF) for obstetric interventions and childbirth events using e-health, and to report completeness and reliability of data collection.

## Methods

### Conceptualization and study sites

A prospective birth cohort study is being conducted including six governmental hospitals in Palestine: Rafidea hospital (Nablus), Palestine Medical Complex (Ramallah) and Hebron Hospital (Hebron) in the West Bank and Shifa Hospital (Gaza city), Shada Al Aqsa (Deir Ballah) and Al Helal Emirati Hospital (Rafah) in Gaza. The birth cohort study also includes an intervention study aiming to prevent perineal tears and will be described elsewhere. The project development phase started in 2013 and the initiative was launched in February 2015. Data collection started on the 1st March 2015 and was scheduled to end on the 30th April 2017.

All governmental hospitals were selected based on their high workload, geographical location and are all teaching sites for physicians, midwives and nurses. Table 1 presents some characteristics of the participating hospitals.

**Table 1:** Characteristics of hospitals

Region	West Bank			Gaza		
Hospital	Rafidia	PMC^b^	Hebron	Shifa	Hilal	Aqsa
Location	Nablus	Ramallah	Hebron	Gaza city	Rafah	Deir AlBalah
No beds^a^	200	231	275	1022	20	280
No maternity beds	43	32	40	80	20	34
Annual births (2014)^a^	6166	4790	6798	12,500	5800	7500
Cesarean section (rate)^a^ (2014)	1724 (28%)	1441 (30.1%)	967 (14.2%)	2000 (16%)	870 (15%)	1020 (13.6%)
No OB/GYN consultants	3	5	8	35	5	16
No residents	17	12	7	42	8	10
No midwives^c^	28	13	21	34	12	20
No nurses^c^	0	0	0	26	10	16
Antenatal care	Yes	Yes	Yes	Yes	Yes	Yes
Operating room	Yes	Yes	Yes	Yes	Yes	Yes
NICU	Yes	Yes	Yes	Yes	Yes	Yes
ICU	Yes	Yes	Yes	Yes	No	Yes
Teaching hospital	Yes	Yes	Yes	Yes	Yes	Yes

^a^Annual report, hospital statistics, OBGYN and midwifery heads in hospitals

^b^PMC: Palestine Medical Complex

^c^These are number of midwives and nurses in the labour and delivery ward

### Study instruments

In summer 2014, the first draft of the CRF for documentation of relevant events during labour and delivery including complications and perineal tears in English was made. The contents of the CRF were based on previous research published from Palestine [21, 22] Norway [18, 19] and the World Health Organization [23, 24] (Additional file 1: presents a summary of the case registration form (CRF) and Additional file 2: The Case Registration Form (CRF) for Palestinian Perineum and Childbirth Study).

### Data collection and entry

Data was collected on all birth events in the six hospitals and registered prospectively by the research teams, mainly by midwives. For this particular study, we used data collected between March 1st and December 31st, 2015. For operational reasons, data registration was restricted to labour and delivery wards; the registration of elective cesarean section was excluded. The reason was simply that women for elective cesarean section are usually admitted to the postpartum or gynecological ward, and are normally cared for by a separate team of midwives and nurses; not included in the initial training for this pilot. All midwives providing intrapartum care for women registered information on all births using the CRF, and the two midwives in each research team entered the data from the CRFs to the DHIS2 system on daily basis or as close as possible to the date of CRF completion. After the completion of data entry, the CRFs were locked up in cupboard prepared in each labor ward beforehand. Only research teams (two midwives) have access to the cupboard. All data were de-identified at point of data entry. Each CRF was given a serial number before data entry that was used to identify the entered CRF. The data was entered into an electronic system that is secured by individual passwords for research team members and investigators. The study was evaluated by the Regional Committee for Medical & Health Research Ethics, Section C, South East Norway, which found the Research Project to be outside the remit of the Act on Medical and Health Research 2008. The project can therefore be implemented without its approval, as long it is in done in accordance with common rules for health care services in Palestine and Norway regarding e.g. privacy.

DHIS2, created by department of global infrastructure at the University of Oslo, was used to collect data. It is worth noting that Palestine is the first country in the Arab World to use DHIS 2 in hospitals, but the system is for the time being also piloted for use in primary health care centers in the West Bank [25].

### Organization and follow up

All healthcare providers working at the labour and delivery wards in the six hospitals were made aware of how to use the CRF form. The CRF form was initiated once the woman was admitted to labour and delivery ward and the data continued to be completed as labour process progressed. Each day the research team collects the completed forms, checks for completeness, enters them into the DHIS2 software, and keeps all forms in folders categorized according to serial numbers for each month.

### Quality assurance methods

To ensure quality (i.e. completeness and reliability) of the collected data, we undertook several measures. In addition to piloting the CRF and training on data registration and data entry, we also monitored the completeness of data collection and entry according to the hospitals’ registries of births and assessed the reliability of data registration and entry in three West Bank hospitals.

Monitoring the completeness of data collection and entry in six hospitals:

The data collection was monitored for completeness by various methods. Weekly field visits were conducted to monitor the process of data registration and identify any difficulties in registration. During field visits, the completed CRFs were reviewed to ensure absence of general and systematic errors in the form and completeness of the variables.

Additionally, a monthly auditing scheme was established to ensure data was collected on all eligible women. Each month, the numbers of registered CRFs in the hospital were cross-checked against the hospital birth register in order to ensure the completeness of data registration. Table 2 shows a summary of the monthly indicators monitored each month in the six hospitals. In order to observe completeness, errors and missing, all births entered in March to July 2015 were investigated for 22 variables using SPSS software. Feedback was given to midwives and research teams accordingly.

**Table 2:** Summary of monthly indicators monitored each month among the six hospitals

Indicator/Hospital	All deliveries	Eligible deliveries for data registration^a^	All completed CRFs	Missing (Deliveries not registered)
PMC	3876	3396	3287 (96.8%)	109 (3.2%)
Rafidia	4284	3603	2593 (72.1%)	1010 (28.0%)
Hebron	5187	4822	4822 (100%)	0
Shifa	11428	10099	9817 (97.2%)	282 (2.8%)
Hilal	4633	4168	4113 (98.7%)	55 (1.3%)
Aqsa	5074	4423	4422 (99.9%)	1 (0.02%)
Totals	34482	30511 (88.5%)	29054 (95.2%)	1457 (4.8%)

^a^All deliveries excluding elective CS (*n =* 3971)

### Assessment of reliability in three West Bank hospitals

Based on our follow up in August 2015, 22 variables were selected to be audited for reliability. The selected variables are presented in Table 3.

**Table 3:** Selected variables for audit

Date of arrival, date of birth, education, number of previous vaginal births, current conditions during this pregnancy, pre-existing medical conditions, number of antenatal visits in this pregnancy, gestational age at arrival, cervical dilation at admission, maternal weight at admission, maternal height, spontaneous/ labor induction, oxytocin augmentation, duration for oxytocin use, duration first stage of labor, duration second stage of labor, delivery method, episiotomy, total number of newborns (this delivery), newborn status at birth, and birth weight.	

A subsample of 80 birth events were audited in each of the three West Bank hospitals between March and December 2015, i.e. a total of 240 birth events. The 80 CRFs from each of the three hospitals were randomly selected. Given the difficulties accessing medical records in the Gaza hospitals, and the fact that Gaza has not yet electronic medical record system; the three hospitals in Gaza were not included in the reliability study. Thus, a subsample comprising of 240 CRFs were randomly selected from the three hospitals in the West Bank between March and December 2015 to be audited. Each birth event was audited for the selected 22 variables (a total of 1760 registrations in each hospital). The 22 variables were registered on a separate form based on information from three sources; the CRF, the DHIS2, and the electronic medical record in the hospital. The audit was conducted over two periods; 30 birth events (660 variables) from March to July, 2015 and 50 birth events (1100 variables) from August to December, 2015. Between the two periods, we communicated the results of the first patch to research teams and midwives in hospitals; particularly the missing and errors in data registration and entry, and encouraged them to improve the processes.

The collected and entered data for each variable was compared against the data documented from the MoH electronic medical record for completeness, similarities and errors. The variables were coded into four categories; correct (variable details are matched between the CRF, DHIS2 and MoH), error (variable details on the CRF or DHIS2 do not match the MoH system), missing (variable details are not documented on the CRF, or DHIS2) and not available (variable details are not available in the MoH system to be crosschecked). However, we compared details of these variables between the CRF and DHIS2. The variables; maternal education, weight, height, and number of antenatal visits were not available in the MoH system. The data was analyzed by IBM SPSS Statistics for Windows (version 21.0, Chicago, IL, USA). The unit of analysis was the variables.

## Results

### Completeness of data registration

**Fig. 1:**
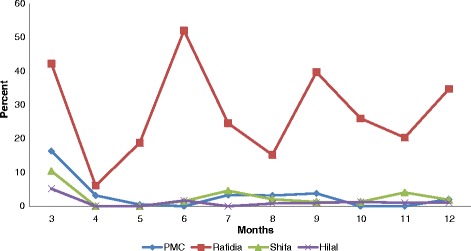
The rate of monthly missing delivery events (not registered) among the six hospitals (March-December, 2015). *The data was completely registered in two hospitals; Hebron and Aqsa

**Fig. 2:**
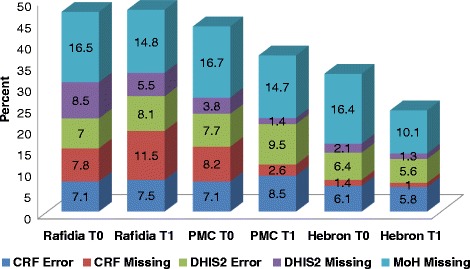
The rates of errors and missing variables in the registered and entered data in the participating hospitals in the West Bank between two periods (March-July and August-Dec., 2016)

A total of 34,482 deliveries occurred between March 1st and December 31st 2015 in the six participating hospitals (Table 2). There were 30,511 (88.5%) eligible birth events to be registered for this study (Table 2). Of all eligible birth events, there were 29,054 (95.2%) completed CRFs. The registration of data was complete in two hospitals (Hebron with (100%) and Aqsa with 99.9%), while the registration of data was less complete at the PMC (96.8%), Hilal Emirati (98.7%), Shifa (97.2%) and Rafidia (72.1%) hospitals (Table 2). Rafidia hospital consistently has the highest proportion of missing birth events registration every month during the data collection period (Fig. 2) (Fig. 1).

In general, as shown in Tables 4 and 5, the rate of the correct (matched between the three sources of information) data registration and entry was high in the three hospitals during the two periods of audit. During the first period of audit, the highest rate of errors in data registration and DHIS was observed at both Rafidia 47 (7.1%), and at the PMC 51 (7.7%), respectively. While the highest rate of missing in data registration and DHIS was observed at the PMC 54 (8.2%) and Rafidia 56 (8.5%), respectively (Table 4). During the second period of audit, the highest rate of errors was observed at the PMC in both data registration 93 (8.5%) and entry 104 (9.5%). While the highest rate of missing was observed at Rafidia in both data registration 127 (11.5%) and DHIS 60 (5.5%) (Table 5). Figure 2 summarizes the rates of errors and missing variables in the registered and entered data for the two periods among the participating hospitals in the West Bank.

**Table 4:** Summary of rates of errors, missing and correct variables (*n =* 660 variable)^a^ in the registered and entered data in the participating hospitals in the West Bank during the first period March-July, 2015

Hospital	CRF (data registration) *N =* 660			DHIS (data entry) *N =* 660			MoH *N =* 660		
	Error N (%)	Missing N (%)	Correct N (%)	Error N (%)	Missing N (%)	Correct N (%)	Error N (%)	Missing N (%)	Correct N (%)
Rafidia	47 (7.1)	52 (7.8)	561 (85)	46 (7)	56 (8.5)	558 (84.5)	1 (0.2)	109 (16.5)	550 (83.3)
PMC	47 (7.1)	54 (8.2)	559 (84.7)	51 (7.7)	25 (3.8)	584 (88.5)	0	110 (16.7)	550 (83.3)
Hebron	40 (6.1)	9 (1.4)	611 (92.6)	42 (6.4)	14 (2.1)	604 (91.5)	0	108 (16.4)	552 (83.6)

^a^30 cases were audited in each hospital for 22 variables (*n =* 660)

**Table 5:** Summary of rates of errors, missing and correct variables (*n =* 1100 variable)^a^ in the registered and entered data in the participating hospitals in the West Bank during the second period August-December 2015

Hospital	CRF (data collection) *N =* 1100			DHIS (data entry) *N =* 1100			MoH *N =* 1100		
	Error N (%)	Missing N (%)	Correct N (%)	Error N (%)	Missing N (%)	Correct N (%)	Error N (%)	Missing N (%)	Correct N (%)
Rafidia	82 (7.5)	127 (11.5)	891 (81)	89 (8.1)	60 (5.5)	951 (86.5)	2 (0.2)	163 (14.8)	935 (85)
PMC	93 (8.5)	29 (2.6)	978 (88.9)	104 (9.5)	15 (1.4)	981 (89.2)	1 (0.1)	162 (14.7)	937 (85.2)
Hebron^b^ *N =* 1034	60 (5.8)	10 (1.0)	964 (93.2)	58 (5.6)	13 (1.3)	963 (93.1)	1 (0.1)	104 (10.1)	929 (89.8)

^a^50 cases were audited in each hospital for 22 variable (*n =* 1100)

^b^In Hebron hospital, 3 files could not be retrieved in the MoH system to be audited. Total audited cases were 47 (*n =* 1034)

As shown in Tables 6 and 7, in both Rafidia and the PMC hospitals, errors in data registration (CRF) and entry (DHIS2) were most frequent for the following variables; age, date of last menstrual period, gestational age, history during current pregnancy, previous medical history, cervical dilation upon admission, and duration of the first stage of labour. The highest percentage of errors in data registration (CRF) and entry (DHIS2) was observed at Rafidia and the PMC was for maternal age (11/29; 37.9% and 11/30; 36.7%) and (8/26; 30.8% and 13/30; 43.3%), respectively. While at Hebron the highest percentage of errors in data registration (CRF) was observed for oxytocin augmentation (7/30; 23.3%) and in the data entry (DHIS2) for current history (5/30; 16.7%).

**Table 6:** The proportions and rates of errors in audited variables in the registered data (CRF audit) in the participating hospitals in the West Bank during the two periods March-July (patch 1) and August-December (patch 2), 2015

Variable	Rafidia			PMC			Hebron		
	N^a^	Errors n (%)	95% CI	N^a^	Errors n (%)	95% CI	N^a^	Errors n (%)	95% CI
Arrival date: patch1	30	1 (3.3)	0.006 to o.167	30	4 (1)	0.035 to 0.256	30	1 (3.3)	0.006 to o.167
Patch 2	50	4 (8)	0.032 to 0.188	49	3 (6.1)	0.021 to 0.1652	47	0	0 to 0.076
Age: patch1	29	11 (37.9)	0.227 to 0.56	30	11 (36.7)	0.219 to 0.545	30	3 (10)	0.035 to 0.256
Patch2	50	7 (14)	0.071 to 0.262	47	9 (19.2)	0.104 to 0.326	46	8 (17.4)	0.091 to 0.307
Education: patch1	24	2 (8.3)	0.023 to 0.258	30	0	0 to 0.114	30	0	0 to 0.114
Patch2	21	0	0 to 0.1546	50	0	0 to 0.071	47	0	0 to 0.076
Previous vaginal Births: patch1	29	3 (10.3)	0.036 to 0.264	27	3 (11.1)	0.039 to 0.281	29	1 (3.5)	0.006 to 0.172
Patch2	48	4 (8.3)	0.033 to 0.196	49	4 (8.2)	0.032 to 0.192	47	7 (14.9)	0.074 to 0.277
Current history:patch1	21	1 (4.8)	0.009 to 0.227	44	0	0 to 0.080	30	5 (16.7)	0.073 to 0.336
Patch2	50	5 (1)	0.044 to 0.214	50	16 (32)	0.208 to 0.458	47	8 (17.0)	0.089 to 0.301
Prev. history:patch1	30	0	0 to 0.114	30	2 (6.7)	0.019 to 0.2133	30	3 (10)	0.035 to 0.256
Patch2	50	3 (6)	0.021 to 0.162	50	18 (36)	0.241 to 0.499	47	9 (19.2)	0.104 to 0.326
No. antenatal visits: patch1	30	0	0 to 0.114	30	0	0 to 0.114	30	0	0 to 0.114
Patch2	44	2 (4.6)	0.013 to 0.151	50	0	0 to 0.071	47	0	0 to 0.076
Last menstrual period: patch1	27	3 (11.1)	0.039 to 0.281	30	10 (33.3)	0.192 to 0.512	29	5 (17.3)	0.076 to 0.346
Patch2	43	7 (16.3)	0.081 to 0.311	44	2 (4.6)	0.013 to 0.151	44	1 (2.3)	0.004 to 0.118
Gestational age: patch1	29	9 (31.0)	0.173 to 0.492	30	1 (3.3)	0.006 to o.167	30	1 (3.3)	0.006 to o.167
Patch2	46	7 (15.2)	0.076 to 0.282	49	1 (2.0)	0.004 to 0.107	46	1 (2.2)	0.004 to 0.113
Cervical dilation on admission: patch1	30	6 (20)	0.095 to 0.373	30	3 (10)	0.035 to 0.256	30	4 (13.3)	0.053 to 0.297
Patch2	48	9 (18.8)	0.102 to 0.319	49	12 (24.5)	0.146 to 0.381	47	4 (8.5)	0.034 to 0.199
Maternal weight: patch1	25	0	0 to 0.133	30	0	0 to 0.114	30	0	0 to 0.114
Patch2	40	0	0 to 0.088	50	1 (2)	0.004 to 0.120	47	0	0 to 0.076
Maternal height: patch1	23	0	0 to 0.143	30	0	0 to 0.114	30	0	0 to 0.114
Patch2	37	0	0 to 0.094	50	0	0 to 0.071	47	0	0 to 0.076
Labor start: patch1	28	0	0 to 0.121	27	0	0 to 0.125	30	2 (6.7)	0.019 to 0.213
Patch2	30	3 (12)	0.042 to 0.311	46	0	0 to 0.077	45	4 (8.9)	0.035 to 0.207
Oxytocin augmentation: patch1	25	3 (12)	0.042 to 0.311	22	2 (9.1)	0.025 to 0.278	30	7 (23.3)	0.118 to 0.409
Patch2	27	3 (11.1)	0.039 to 0.281	45	4 (8.9)	0.035 to 0.207	46	5 (17.9)	0.079 to 0.356
Augmentation Duration: patch1	25	3 (12)	0.042 to 0.311	21	3 (14.3)	0.0498 to 0.346	28	5 (17.9)	0.079 to 0.356
Patch2	43	5 (11.6)	0.051 to 0.245	49	1 (2.0)	0.004 to 0.107	47	2 (4.3)	0.012 to 0.143
Duration 1st stage: patch1	30	0	0 to 0.114	29	3 (10.3)	0.036 to 0.264	28	0	0 to 0.121
Patch2	44	9 (20.5)	0.112 to 0.35	49	8 (16.3)	0.085 to 0.290	45	8 (17.8)	0.093 to 0.313
Duration 2nd stage: patch1	30	1 (3.3)	0.006 to o.167	29	1 (3.5)	0.006 to 0.172	30	1 (3.3)	0.006 to o.167
Patch2	49	5 (11.6)	0.051 to 0.245	40	5 (12.5)	0.055 to 0.2611	47	0	0 to 0.076
Mode of delivery: patch1	29	1 (3.5)	0.006 to 0.172	30	0	0 to 0.114	30	0	0 to 0.114
Patch2	50	0	0 to 0.071	50	1 (2)	0.004 to 0.11	47	1 (2.1)	0.004 to 0.111
Episiotomy: patch1	26	0	0 to 0.129	30	1 (3.3)	0.006 to o.167	30	0	0 to 0.114
Patch2	44	4 (9.1)	0.036 to 0.212	49	3 (6.1)	0.021 to 0.165	47	0	0 to 0.076
No. newborns: patch1	30	0	0 to 0.114	30	0	0 to 0.114	28	0	0 to 0.121
Patch2	48	3 (6.3)	0.022 to 0.168	50	1 (2)	0.004 to 0.11	47	0	0 to 0.076
Status at birth: patch1	29	0	0 to 0.117	29	0	0 to 0.117	29	0	0 to 0.117
Patch2	46	0	0 to 0.077	49	1 (2.0)	0.004 to 0.107	47	0	0 to 0.076
Birth weight: patch1	29	3 (10.3)	0.036 to 0.264	30	3 (12)	0.042 to 0.311	30	2 (6.7)	0.019 to 0.213
Patch2	50	2 (4)	0.011 to 0.135	50	3 (6)	0.021 to 0.162	47	2 (4.3)	0.012 to 0.143

The number of cases during the first patch = 30, second patch = 50

^a^ *N =* number of selected cases in each hospital

**Table 7:** The proportions and rates of errors variables in the registered data (DHIS2 audit) in the participating hospitals in the West Bank during the two periods March-July (patch 1) and August-December (patch 2), 2015

Variable	Rafidia			PMC			Hebron		
	N^a^	Errors n (%)	95% CI	N^a^	Errors n (%)	95% CI	N^a^	Errors n (%)	95% CI
Arrival date: patch1	29	2 (6.9)	0.019 to 0.221	30	4 (13.3)	0.053 to 0.297	29	1 (3.5)	0.006 to 0.172
Patch 2	50	6 (12)	0.056 to 0.238	50	2 (4)	0.011 to 0.135	47	1 (2.1)	0.004 to 0.111
Age: patch1	26	8 (30.8)	0.165 to 0.511	30	13 (43.3)	0.274 to 0.608	30	6 (2)	0.095 to 0.373
Patch2	49	8 (16.3)	0.085 to 0.290	50	12 (24)	0.143 to 0.374	47	7 (14.9)	0.074 to 0.277
Education: patch1	23	2 (8.7)	0.024 to 0.268	30	0	0 to 0.114	30	0	0 to 0.114
Patch2	40	0	0 to 0.0876	49	0	0 to 0.0727	47	0	0 to 0.076
Previous vaginal Births: patch1	29	3 (10.3)	0.036 to 0.264	29	3 (10.3)	0.036 to 0.264	29	1 (3.5)	0.006 to 0.172
Patch2	50	5 (0.1)	0.044 to 0.214	50	4 (8)	0.032 to 0.188	47	5 (10.6)	0.046 to 0.226
Current history:patch1	28	1 (3.6)	0.006 to 0.177	30	3 (1)	0.035 to 0.256	30	5 (16.7)	0.073 to 0.336
Patch2	50	6 (12)	0.056 to 0.238	50	16 (32)	0.208 to 0.458	47	7 (14.9)	0.074 to 0.277
Prev. history:patch1	29	1 (3.5)	0.006 to 0.172	30	1 (3.3)	0.006 to o.167	30	4 (13.3)	0.053 to 0.297
Patch2	50	3 (6)	0.021 to 0.162	50	18 (36)	0.241 to 0.499	47	7 (14.9)	0.074 to 0.277
No. antenatal visits: patch1	29	0	0 to 0.117	30	0	0 to 0.114	30	0	0 to 0.114
Patch2	49	3 (6.1)	0.021 to 0.165	48	2 (4.2)	0.012 to 0.141	47	0	0 to 0.076
Last menstrual period: patch1	26	3 (11.5)	0.04 to 0.291	29	10 (34.5)	0.199 to 0.527	26	2 (7.7)	0.021 to 0.241
Patch2	43	7 (16.3)	0.081 to 0.311	47	6 (12.8)	0.161 to 0.252	44	1 (2.3)	0.004 to 0.118
Gestational age: patch1	29	9 (31.0)	0.173 to 0.492	29	2 (6.9)	0.019 to 0.221	30	3 (0.1)	0.035 to 0.256
Patch2	50	9 (18)	0.098 to 0.308	50	2 (4)	0.011 to 0.135	47	1 (2.1)	0.004 to 0.111
Cervical dilation on admission: patch1	29	6 (20.7)	0.099 to 0.384	30	2 (6.7)	0.019 to 0.213	29	3 (10.3)	0.036 to 0.264
Patch2	49	10 (20.4)	0.115 to 0.336	49	10 (20.4)	0.115 to 0.336	46	5 (10.9)	0.047 to 0.230
Maternal weight: patch1	24	0	0 to 0.138	30	0	0 to 0.114	30	1 (3.3)	0.006 to o.167
Patch2	40	1 (2.5)	0.004 to 0.129	50	2 (4)	0.011 to 0.135	47	1 (2.1)	0.004 to 0.111
Maternal height: patch1	22	0	0 to 0.149	30	0	0 to 0.114	29	0	0 to 0.117
Patch2	38	0	0 to 0.092	50	2 (4)	0.011 to 0.135	47	2 (4.3)	0.012 to 0.143
Labor start: patch1	28	0	0 to 0.121	27	2 (7.4)	0.021 to 0.234	29	2 (6.9)	0.019 to 0.221
Patch2	49	5 (10.2)	0.044 to 0.218	49	2 (4.1)	0.011 to 0.137	47	1 (2.1)	0.004 to 0.111
Oxytocin augmentation: patch1	27	5 (18.5)	0.082 to 0.367	22	2 (9.1)	0.025 to 0.278	30	6 (20)	0.095 to 0.373
Patch2	42	3 (7.1)	0.025 to 0.190	50	4 (8)	0.032 to 0.188	47	5 (10.6)	0.046 to 0.226
Augmentation Duration: patch1	25	3 (12)	0.042 to 0.311	22	3 (13.6)	0.048 to 0.333	29	4 (13.8)	0.055 to 0.306
Patch2	45	4 (8.9)	0.035 to 0.207	50	1 (2)	0.004 to 0.105	47	2 (4.3)	0.012 to 0.143
Duration 1st stage: patch1	29	0	0 to 0.117	29	3 (10.3)	0.036 to 0.264	27	0	0 to 0.125
Patch2	45	10 (22.2)	0.125 to 0.363	50	10 (20)	0.112 to 0.330	45	11 (24.4)	0.142 to 0.387
Duration 2nd stage: patch1	29	1 (3.5)	0.006 to 0.172	29	1 (3.5)	0.006 to 0.172	29	1 (3.5)	0.006 to 0.172
Patch2	50	6 (12)	0.056 to 0.238	50	5 (10)	0.044 to 0.214	47	1 (2.1)	0.004 to 0.111
Mode of delivery: patch1	29	0	0 to 0.117	30	0	0 to 0.114	30	1 (3.3)	0.006 to o.167
Patch2	49	0	0 to 0.073	50	0	0 to 0.071	47	0	0 to 0.076
Episiotomy: patch1	28	0	0 to 0.121	30	1 (3.3)	0.006 to o.167	30	0	0 to 0.114
Patch2	42	0	0 to 0.084	50	2 (4)	0.011 to 0.135	47	0	0 to 0.076
No. newborns: patch1	29	0	0 to 0.117	30	0	0 to 0.114	30	0	0 to 0.114
Patch2	50	0	0 to 0.071	50	0	0 to 0.071	47	0	0 to 0.076
Status at birth: patch1	29	0	0 to 0.117	29	0	0 to 0.117	30	0	0 to 0.114
Patch2	50	1 (2)	0.004 to 0.11	50	1 (2)	0.004 to 0.11	47	0	0 to 0.076
Birth weight: patch1	28	2 (7.1)	0.021 to 0.226	30	1 (3.3)	0.006 to o.167	30	2 (6.7)	0.019 to 0.213
Patch2	50	2 (4)	0.011 to 0.135	50	3 (6)	0.021 to 0.162	47	1 (2.1)	0.004 to 0.111

The number of cases during the first patch = 30, second patch = 50

^a^ *N =* number of selected cases in each hospital x number of audited variables

Missing of registration and entry data were, however, more frequent in the following variables; history of current pregnancy, education, maternal weight, maternal height, oxytocin augmentation and augmentation duration as shown in the proportions (N) in Tables 6 and 7. The highest rate of missing in data registration (CRF) in Rafidia was observed for maternal education (29/50; 58%), at the PMC data on augmentation duration (9/30; 30%), and at Hebron hospital data on last menstrual period (6/50; 12%) Table 6. The highest rate of missing in data entry (DHIS2) was observed at Rafidia for maternal height (8/30; 26.7%), at the PMC for the variable on oxytocin augmentation and duration (8/30; 26.7%), and at Hebron hospital for the variable of the last menstrual period (4/30; 13.3%) Table 7.

As shown in Fig. 2, improvements were observed given decreasing rates of missing variables in data registration and data entry in the three hospitals between the two periods of audit, except for Rafidia hospital (i.e., the rate of missing variables in the CRFs increased; 7.8% to 11.5%). No improvement was observed in the rate of errors of variable registration and entry in the three hospitals, except for Alia hospital in Hebron (i.e., the rate of errors in data entry was slightly decreased; 6.1% to 5.8%).

Information on education, the number of antenatal care visits, maternal weight and maternal height, were not available in the MoH electronic information system, and thus, the comparison for completeness and quality was conducted between the CRF and the DHIS.

A total of 16 selected cases (eight from Rafidia, four from the PMC, and four from Hebron) could not be retrieved from the Ministry electronic information system to do the audit due to errors or insufficient registration of an identification number (i.e., ID or file number).

## Discussion

To our knowledge this is the first birth cohort study using e-health in Palestine. The main finding was that the registered and entered data on birth events was almost complete in five out of six hospitals during the first nine months of the study. Among 80 birth events audited for 22 variables (i.e. 1760 registrations) in three hospitals in the West Bank, the rates for correct data registration ranged from 81% to 93.2% and for data entry 84.5% to 93.1%.

A major strength of this study is the large and detailed data set on maternal and newborn health including governmental hospitals in Gaza and West Bank simultaneously. The available data from MOH, PCBS and individual hospital-based studies are useful to give a general understanding of women’s health status in Palestine,, but not sufficient to understand and evaluate the specific processes, practices, or standards of care, which makes evaluating quality a very challenging task and thus, improvements are slow or even not possible to be recommended or integrated. [7, 8, 22]. The advantage of our study is the vast information on maternal newborn health before and during labour, which will enable us to adjust for possible confounding factors when analyzing our data. A weakness, however, may be the fact the hospitals in Gaza do not have electronic medical records and were therefore excluded from the audit. We therefore conducted a separate reliability study for data collected in the Gaza hospitals that will be published elsewhere. Another limitation might be related to small subsamples of cases in each hospital. However, the selection of these cases was random, the audit process included 22 variables from three different sources and the unit of analysis was variables.

Although medical and birth registries have been successfully implemented in many countries, challenges have been reported [4–6, 17]. Similar to other countries, mainly midwives are responsible for data registration [4, 18] and entry to the DHIS2. This fact may have added to the completeness of our data, since midwives in some countries are more used to register data on routine than medical doctors. Our CRF included more information than the routine process of documentation in the participating hospitals and therefore added extra workloads for the midwives, which may explain the incomplete registration in some hospitals. It may also have weakened the accuracy on pre-pregnancy diseases, concomitant diseases or delivery-related conditions such as perineal tears, since physicians are responsible to set diagnosis and most of them were not engaged to register data.

Integrating technology into a health care system can be a complex process, particularly in low and middle income countries LMICs. Boonstra et al. identified many reasons for resistance in physicians to use i.e. electronic Medical Records [26]. In our study, the lack of participation of physicians in the data registration and entry could be related to the lack of incentives, lack of leadership and a perception that this would be time consuming or negatively influence professional autonomy, which is consistent with the analysis of barriers behind the resistance of physicians to integrate technology into healthcare [26]. In our study, physicians in governmental hospitals in Gaza may have lacked motivation to assist the data registration due to the fact that they do not receive salaries on a monthly basis. In the West Bank, the physicians are paid salaries on a regular basis, but are not used to contribute to academic work or studies. Anyhow, medical doctors also have to focus on non-obstetric patients, giving them fewer opportunities to contribute to the study. Despite this, the registered and entered data was more comprehensive and of better quality than the data found in the MoH electronic system.

While data registration was complete in Hebron and Aqsa hospitals, the proportion of incomplete registration of birth events was consistently high in Rafidia hospital compared to the PMC, Shifa and Hilal hospitals. This is perhaps due to the lack of motivation observed among the staff in Rafidia hospital since the beginning of the project due to changes in the department’s leadership. On the other hand, it is not surprising that the best quality of data registration and entry was at Hebron hospital as the data has the lowest rates in missing and errors in both registration and entry as the motivation among of the team was observed to be high. Previous studies have reported that incomplete data registration is common in high-income as well as from low-income countries, when it comes to sensitive data related to maternal deaths, and the major complications during childbirth such as hemorrhage, hypertensive disease and sepsis [5, 27, 28].

In spite of our thorough, detailed and documented feedback that was given to our research teams and midwives in the three hospitals in September about the specific variables where missing / errors were found, little improvement was observed. Although there is minimal overall improvement in variable registration and entry noticed between the two periods in the three hospitals, it is worth to mention that the most frequent errors in data registration were in different variables in each hospital between the two periods. Improvement was observed in both data registration and entry between individual variables among all hospitals evident by decrease in the number of errors or missing between the two periods. Despite that, data registration can be described as an excellent kickoff and the data can be considered satisfactory reliable. For example, the percentage of missing data for the variables gestational age and birth weight on the DHIS2 at our three hospitals was only 2.1% (5 cases/237 audited cases) for each variable, compared to 0.6% and 0.02% missing data on the same variables respectively reported from Norway [19]. The literature from the Nordic countries confirmed that their data registration has substantially improved overtime and currently the registered data in the Nordic birth registries are highly accurate for most variables [19].

From our continuous follow up, we may suggest that the observed improvements in the missing rates of data registration and entry in the three hospitals were mainly due to the extra efforts of research teams who put more efforts to revise and go back to the ministry electronic information system to complete any missing data in the CRFs before entry as possible. Additionally, new midwifery staff has replaced almost half of the midwives between November and December 2015, which may have contributed to the lack of satisfactory improvement. However, the new staff were coached and trained on the CRF registration by our research team and midwives in the three hospitals, which may indicate the sense of ownership to the registration process by research teams in the hospitals.

The initiation of data registration and entry in the Palestinian hospitals faced some challenges. The registration process was viewed by healthcare providers (physicians and midwives) as a process requiring financial incentive, which has negatively influenced the physician’s active participation in the process and could be a challenge to sustain the process in the future. Moreover, some data could not be registered in a timely manner. The CRF could be completed after the occurrence of the birth events which may have contributed to errors or missing in registration as midwives are usually too few and too busy each shift. For the non-registered birth events, our research team usually tried to retrieve their information from the Ministry electronic system where not all information may be accessed and some variables are not even available, such as education. And finally, it was not possible to search the DHIS2 for certain birth event by the identification number, which was difficult and time consuming for the researcher to retrieve entered information about birth events and compare it with other sources to check reliability.

Over and above, this analysis may also have the limitation that it examines a small number of birth events compared to the total number of birth occurred in the three hospitals. However, we audited 22 variables and thus, we believe that this exercise has succeeded to shed some light on the quality of registered and entered data in the three hospitals in the West Bank and the needs for improvement.

To improve the quality of maternal and newborn healthcare in Palestinian hospitals, complete and reliable data on the details of significant birth events are essential. The medical birth registry serves as a valuable tool for monitoring, quality assurance and research. The basis for this tool was laid in six Palestinian hospitals. The smart integration of this tool into the current Palestinian health care system is an opportunity that shall assist to identify specific areas of substandard care during childbirth, reinforce the implementation of clinical guidelines into daily practice, implement further interventions and strategies for improvement, detect new diseases, and open valuable opportunities for research.

## Conclusions

A combination of registration (CRF) and entry (DHIS2) systems for the details of childbirth events and interventions using e-health were successfully implemented and the process of registration is currently rolled out in six Palestinian hospitals. A series of scientific publications on different areas of obstetrics interventions and complications during childbirth are expected to be published in the near future using this huge first set of data set from Palestine. The registered and entered data on birth events in six hospitals was almost complete in five out of six hospitals. The collected data is considered reliable for research purposes.

## Additional files

Additional file 1: Summary of the case registration form (CRF). (DOCX 15 kb)

Additional file 2: CRF Palestinian Perineum study. Palestinian Perineum and Childbirth Study. The research tool. (PDF 1192 kb)

## Abbreviations

CRF: Case registration form used for data registration
DHIS2: District Health Information Software 2
EMRs: Electronic Medical Records
HISP: Health Information Systems Programme
LMIC: Low and Middle Income Countries
MDGs: Millennium Development Goals
MICS: Multiple Indicators Cluster Survey
MoH: Ministry of Health
PCBS: The Palestinian Central Bureau of Statistics
PMC: Palestine Medical Complex
WHO: World Health Organization
