# Supplementary material for: Building a research registry for studying birth complications and outcomes in six Palestinian governmental hospitals
Source: BMC Pregnancy Childbirth. 2017 Apr 11;17:112. doi: 10.1186/s12884-017-1296-6 (PMC5387267; doi:10.1186/s12884-017-1296-6)
Supplement: Supplementary file 1 — Summary of the case registration form (CRF). (DOCX 15 kb) [file 12884_2017_1296_MOESM1_ESM.docx]

Additional file 1: Summary of the case registration form (CRF)

| **Major field and Variables** | |
| --- | --- |
| **General information and Arrival to hospital**  Patient name  Patient ID number; phone number  Hospital  Date and time of arrival  Birth attendant | **Background information**  Date of birth  Marital status  Marriage between first cousins  Education, total years at school and studying  Place of residence  Prepregnancy maternal weight  Maternal weight at admission  Maternal height  Smoking |
| **Previous pregnancies (excluding current pregnancy)**  Number of previous vaginal births  Number of children alive  Number of previous caesareans  Number of trimester abortions  Number of ectopic pregnancies  Pre-existing medical conditions | **Maternal health in the current pregnancy (before labour)**  Last menstruation period  Number of antenatal visits in this pregnancy  IVF  Ultrasound estimated date of birth  Current conditions during this pregnancy  Mother reports medication she has used during pregnancy |
| **Reason for arrival**  Reason for arrival  Gestational age at arrival  Cervical dilation at admission  Blood pressure at arrival  Urine test  From CBC at admission | **Labour start**  Partogram Present  Spontaneous/Labour induction  Indication for induction  Induction method  Amniotic fluid color  Oxytocin augmentation  Oxytocin in dropper  Indication for oxytocin use  Duration for oxytocin use  Duration first stage of labor  Duration second stage of labor  Duration of active second stage of labour |
| **Birth/delivery**  Pain relief  Medication during labor  Complications during labor  Delivery method  Indication for operative delivery  Episiotomy  Indication for episiotomy  Perineal tears  Perineal tear/episiotomy sutured by  Vaginal ephitelum sutured  Perineal muscles sutured  Perineal skin sutured  Total number of newborns (this delivery)  Date of delivery  Time of delivery  Fetal presentation at birth  Newborn status at birth  Admission NICU  Birthweight  Gender  Apgar score 5 min/Apgar 10 min  Newborn has malformation | **Postpartum/third stage of labour**  Prophylactic oxytocin/other  Excessive bleeding  Treatment for excessive bleeding  Placenta  Placenta inspection  Blood transfusion  Uterine rupture  Hysterectomy  Admission of mother to intensive care unit  Amission ucome  Time point for discharge |
