# Supplementary material for: Building a research registry for studying birth complications and outcomes in six Palestinian governmental hospitals
Source: BMC Pregnancy Childbirth. 2017 Apr 11;17:112. doi: 10.1186/s12884-017-1296-6 (PMC5387267; doi:10.1186/s12884-017-1296-6)
Supplement: Supplementary file 2 — CRF Palestinian Perineum study. Palestinian Perineum and Childbirth Study. The research tool. (PDF 1192 kb) [file 12884_2017_1296_MOESM2_ESM.pdf]

Oslo University Hospital

Palestinian Perineum and Childbirth Study

Do NOT fill in this box – for research team only.

1. Patient: Last name ________ First name ________

2. Patient ID number: ________ **Phone number 1:** ________ **Phone number 2:** ________

3. Hospital: ☐ Al Helal Emirati ☐ Queen Alia ☐ PMC ☐ Rafidia ☐ Shifa ☐ Shada Al Aqsa

Arrival to hospital

4. Date and time of arrival: dd | mm | yy Time (24 hour) __ : __

5. Birth attendant: MW=1 OBGYN=2 Student=3 Resident=4

Background information

7. Date of birth: dd | mm | yy

8. Marital status: ☐ Married ☐ Other ☐ Separated/widowed

9. Marriage between first cousins: ☐ No ☐ Yes

10. Education, total years at school and studying: ____

11. Place of residence: ☐ Urban ☐ Rural ☐ Camp

12. Prepregnancy maternal weight: ____ Kg

13. Maternal weight at admission: ____ Kg

14. Maternal height: ____ Cm

15. Smoking (cigarettes/arghila): ☐ No ☐ Yes

Previous pregnancies (excluding current pregnancy)

16. Number of previous vaginal births (> 23+6): ____

Of these, how many forceps or vacuum deliveries? ____

17. Number of children alive: ____

18. Number of previous caesareans: ____

19. Number of trimester abortions: 1st ____ 2nd ____

20. Number of ectopic pregnancies: ____

21. Pre-existing medical conditions: ☐ Hypertension ☐ Diabetes ☐ Anaemia ☐ Hypothyroidism ☐ Other: ________

Maternal health in the current pregnancy (before labour)

22. Last menstruation period: dd | mm | yy

23. Number of antenatal visits in this pregnancy: ____

24. IVF: ☐ No ☐ Yes

25. Ultrasound estimated date of birth: dd | mm | yy

26. ☐ Gestational hypertension ☐ Pre-eclampsia ☐ Diabetes ☐ Gestational diabetes

Other: ________

27. Mother reports medication she has used during pregnancy: ☐ None ☐ Antihypertensive medication ☐ Anticoagulants ☐ Pain killers ☐ Iron ☐ Vitamin supplement ☐ Folic acid ☐ Other If yes: Please write name of medication and dose:

____

Reason for arrival to hospital

28. ☐ Contractions ☐ ROM ☐ Abdominal pain ☐ Vaginal bleeding ☐ PE/hypertensive disorder ☐ Eclampsia Other: ________

29. Gestational age at arrival: ____ Weeks

30. Cervical dilatation at admission: ____ Cm

31. Blood pressure at arrival: ____ mm HG

32. Urine test: ____

33. From CBC at admission: ____ HB ____ Hct ____ Platelets ____ White blood cells

Palestinian Perineum and Childbirth Study

Do NOT fill in this box – for research team only.

Labour start

34. **Partogram present:** ☐ No ☐ Yes

35. ☐ Spontaneous (*if yes; please go to question 37*)
☐ Labour induction (*if yes; please continue to question 36*)

36. **Indication for induction:** ☐ PROM
☐ Reduced fetal movement ☐ Post term pregnancy
☐ Hypertensive disorder ☐ Diabetes
☐ Fetal growth restriction ☐ Large baby

37. **Induction method:** ☐ Balloon catheter ☐ Amniotomy
☐ Cytotec/Misoprostol ☐ Prostin ☐ Oxytocin

38. **Amniotic fluid colour:**
☐ Normal ☐ Meconium stained ☐ Blood stained

39. **Oxytocin augmentation:**
☐ No (*if no; please go to question 43*)
☐ Yes (*If yes; please go to question 40*)

40. **Oxytocin in dropper**
☐ No ☐ Yes

41. **Indication for Oxytocin use:**
☐ Prolonged first stage
☐ Prolonged second stage

42. **Duration for Oxytocin use:** Hours ☐ Minutes ☐

43. **Duration first stage of labour** (HOURS): ☐

44. **Duration second stage of labour** (MINUTES): ☐

45. **Duration of active second stage of labour** (pushing): ☐

Birth/delivery

46. **Pain relief:** ☐ None ☐ Opioids (Pethidine®) ☐ Analgesics
☐ Pudendal ☐ Paracervical block ☐ Epidural ☐ Spinal
☐ Local infiltration anesthesia ☐ General anesthesia

47. **Medication during labour:** ☐ No ☐ Yes
If yes: ☐ Insulin ☐ Magnesium Sulfate ☐ Anticoagulants
☐ Other If other please specify:
☐

48. **Complications during labour:** ☐ None ☐ Fever
☐ Convulsions ☐ Hypertensive crisis ☐ Bleeding
☐ Other If other please specify:
☐

49. **Delivery method:** ☐ Spontaneous ☐ Vacuum extraction
☐ Forceps ☐ Emergency caesarean ☐ Planned caesarean

50. **Indication for operative delivery:**
☐ Fetal distress/abnormal CTG ☐ Obstructed labour
☐ Multiple gestations ☐ Eclampsia
☐ Maternal hypertension/preeclampsia ☐ Bleeding
☐ Maternal exhaustion ☐ Previous cesarean
☐ Malpresentation ☐ Other If other please specify:
☐

51. **Episiotomy:** ☐ No ☐ Yes

52. **Indication for episiotomy:** ☐ Fetal distress
☐ Protecting perineum ☐ Primiparity
☐ Instrumental delivery ☐ Prolonged second stage

53. **Perineal tears:** ☐ Intact perineum ☐ First degree tear
☐ Second degree tear ☐ Third degree tear ☐A ☐B ☐C
☐ Fourth degree tear

54. **Perineal tear/episiotomy sutured by:** ☐ Midwife ☐ OBGYN

55. **Vaginal ephitelum sutured:**
☐ Continous ☐ Interrupted ☐ Subcuticular

56. **Perineal muscules sutured:**
☐ Continous ☐ Interrupted ☐ Subcuticular

57. **Perineal skin sutured:**
☐ Continous ☐ Interrupted ☐ Subcuticular

58. **Total number of newborn (this delivery)**[1]**:** ☐

59. **Date of delivery** (dd/mm/yy)[1]: ☐ ☐ ☐

60. **Time of delivery** (24 hour format)[1]: ☐ : ☐

61. **Fetal presentation at birth**[1]**:** ☐ Normal cephalic
☐ Occiput posterior ☐ Breech ☐ Others

62. **Newborn at birth**[1]**:**
☐ Alive ☐ Stillbirth

63. **Admission NICU**[1]**:**
☐ No ☐ Yes

64. **Birthweight**[1]**:** (GRAMS): ☐

65. **Gender**[1]**:** ☐ Girl ☐ Boy

66. **Apgar score 5 min**[1]**:** ☐ **Apgar 10 min**[1]**:** ☐

67. **Newborn has malformation**[1]**:**
☐ No ☐ Yes, major ☐ Yes, minor malformation
☐

Postpartum/third stage of labour

68. ☐ Prophylactic oxytocin i.m. ☐ Other
☐

69. **Excessive bleeding (>500 ml):** ☐ No ☐ Yes

70. **Treatment for excessive bleeding:**
☐ Methergin ☐ Cytotec/Misoprostol
☐ Oxytocin i.v. ☐ Other If other please specify:
☐

71. **Placenta:** ☐ Separates spontaneously ☐ Crede maneuver
☐ Controlled cord traction ☐ Manual removal

72. **Placenta inspection:** ☐ Normal ☐ Velamentous
☐ Placenta accreta ☐ Placenta percreta

73. **Blood transfusion:** ☐ Yes ☐ No

74. **Uterine rupture:** ☐ No ☐ Yes

75. **Hysterectomy:** ☐ Yes ☐ No

76. **Admission of mother to intensive care unit:** ☐ No ☐ Yes

77. **Admission outcome:** ☐ Discharged
☐ Referred to other hospital ☐ Maternal death

78. **Time point for discharge:** ☐ ☐ ☐
dd | mm | yy

Oslo University Hospital

[1] By multiple gestation, please use one sheet for each child for question 58 to 67.

Signature from the person filling out this form. Write name with capital letters.
